# A Fast Association Test for Identifying Pathogenic Variants Involved in Rare Diseases

**DOI:** 10.1016/j.ajhg.2017.05.015

**Published:** 2017-06-29

**Authors:** Daniel Greene, NIHR BioResource, Sylvia Richardson, Ernest Turro

**Affiliations:** 1 Department of Haematology, University of Cambridge, Cambridge Biomedical Campus, Cambridge CB2 0XY, UK; 2 NHS Blood and Transplant, Cambridge Biomedical Campus, Cambridge CB2 0PT, UK; 3 Medical Research Council Biostatistics Unit, Cambridge Biomedical Campus, Cambridge CB2 0SR, UK

**Keywords:** rare diseases, Mendelian diseases, hereditary disorders, rare variants, rare variant association test, Bayesian inference, whole-genome sequencing

## Abstract

We present a rapid and powerful inference procedure for identifying loci associated with rare hereditary disorders using Bayesian model comparison. Under a baseline model, disease risk is fixed across all individuals in a study. Under an association model, disease risk depends on a latent bipartition of rare variants into pathogenic and non-pathogenic variants, the number of pathogenic alleles that each individual carries, and the mode of inheritance. A parameter indicating presence of an association and the parameters representing the pathogenicity of each variant and the mode of inheritance can be inferred in a Bayesian framework. Variant-specific prior information derived from allele frequency databases, consequence prediction algorithms, or genomic datasets can be integrated into the inference. Association models can be fitted to different subsets of variants in a locus and compared using a model selection procedure. This procedure can improve inference if only a particular class of variants confers disease risk and can suggest particular disease etiologies related to that class. We show that our method, called BeviMed, is more powerful and informative than existing rare variant association methods in the context of dominant and recessive disorders. The high computational efficiency of our algorithm makes it feasible to test for associations in the large non-coding fraction of the genome. We have applied BeviMed to whole-genome sequencing data from 6,586 individuals with diverse rare diseases. We show that it can identify multiple loci involved in rare diseases, while correctly inferring the modes of inheritance, the likely pathogenic variants, and the variant classes responsible.

## Introduction

Hundreds of thousands of individuals with rare disorders are undergoing whole-genome sequencing in an effort to identify novel disease etiologies, increase our understanding of biological processes, and improve clinical care.^1^ Thanks to the affordability of DNA sequencing, population association study designs for diseases affecting fewer than 1 in 2,000 people are now possible. However, the statistical association methods required to identify relevant loci need to fulfil several criteria in order to be well-powered, particularly when the number of cases with a particular disease is small. First, they need to allow some sharing of information across variants because rare diseases are often genetically heterogeneous. Second, they need to account for the presence of pathogenic rare variants that act upon disease risk in a dominant or a recessive manner alongside benign rare variants that do not affect disease risk. Third, they must be capable of integrating prior information into the inference regarding the plausibility of a locus being implicated in a disease and variant-level co-data on pathogenicity. Such co-data can be derived from population allele frequency databases, consequence predictions, conservation-based predictions, or genomic datasets, for example. Lastly, methods need to have efficient implementations that enable fast application across a large number of regions in the genome.

Frequentist association tests for rare variants include the Burden test and the sequence kernel association test (SKAT).^2^ The Burden test regresses the phenotype on a genetic score obtained by summing allele counts across all rare variants in a locus. The cohort allelic sums test (CAST)^3^ uses a genetic score that is equal to 1 if an individual harbors at least 1 (or 2) variants under a dominant (or recessive) inheritance model, and 0 otherwise, but is statistically equivalent to the Burden test in other respects. SKAT specifies a random effect for each variant and performs a score test under the null hypothesis that the variance of the random effects is zero. The variance-covariance structure of the random effects under the alternative hypothesis is determined by a kernel function, which would typically be a weighted genetic correlation across the variants in the locus. SKAT can incorporate nuisance covariates, accounts for linkage disequilibrium between variants under consideration, and is well-powered for traits whereby many different variants in a locus with varying effect sizes and allele frequencies contribute to the phenotype.

For scientific follow-up, it is important to infer which variants are likely to be pathogenic, conditional on an association being present in a given locus. The backward elimination^4^ procedure removes individual variants iteratively from the predictors as long as this increases a test statistic of association (either Burden or SKAT). The adaptive combination of p values (ADA)^5^ algorithm ranks variants by p value obtained using Fisher’s exact test and selects a threshold on p value that maximizes an aggregate test statistic. As these algorithms prune variants in a stepwise fashion, they do not explore the full space of possible combinations of pathogenic variants. It is also important that inference can be performed sufficiently quickly to enable applications across tens of thousands of regions, with tens to hundreds of variants in each one. The methods above, however, rely on permutations to obtain empirical p values, rendering them computationally expensive.

In principle, Bayesian inference lends itself well to rare variant association analysis because it provides a coherent framework for sharing information across variants and provides a natural way of incorporating prior information on variant pathogenicity. The variational Bayes discrete mixture method (vbdm),^6^ the Bayesian risk index,^7^ and the Bayesian rare variant detector (BRVD)^8^ all model a mixture of pathogenic and non-pathogenic variants in a locus, but they employ additive models of disease risk or severity more suited to complex rather than rare diseases caused by dominant or recessive inheritance of one or two pathogenic alleles.

Here we present a Bayesian model in which disease risk depends on the genotypes at rare variants in a locus, a latent mode of inheritance, and a latent partition of variants into pathogenic and non-pathogenic subsets. Different modes of inheritance are modeled by conditioning the probability of case status on the number of pathogenic alleles and the ploidy for each individual at the variants. Thus, disease risk due to compound heterozygosity or X-linked inheritance is explicitly accommodated. Prior knowledge concerning variant pathogenicity can be incorporated in the form of shifts in the log odds of pathogenicity relative to a global mean. By placing a vague prior distribution on the scale of these shifts, the usefulness or otherwise of these co-data are accounted for flexibly to maximize power.

For a given set of variants, inference is performed by comparing the model described above with a baseline model in which disease risk is independent of the genotypes. The mode of inheritance and the pathogenicity of each variant, conditional on an association, can be inferred through the posterior distributions of parameters in the model. Particular classes of variants in a locus may be the only ones that confer disease risk. For example, only variants in the 5′ UTR region or only high-impact coding variants may be involved. Our method can compare models fitted to different classes of variants in order to infer which ones are responsible for disease. Typically the inference process would be repeated over many sets of variants selected from different loci throughout the genome. The procedures are implemented in an efficient R package called BeviMed, which stands for Bayesian evaluation of variant involvement in Mendelian disease.

## Material and Methods

### Model Specification

Let *y* be a binary vector of length *N* indicating whether individual *i* is a case (*y*_*i*_ = 1) or a control (*y*_*i*_ = 0) subject with respect to a particular disease. Suppose *k* rare variants are under consideration (typically in a particular genomic region) and the genotype for individual *i* at variant *j* is coded in the *i*th row and *j*th column of the genotype matrix *G*. A genotype of 0 or 2 denotes homozygosity for the common or minor allele, respectively, and a genotype of 1 denotes heterozygosity. Under a baseline model, labeled *γ* = 0, *y* is independent of *G* and all individuals have a probability of being a case *τ*_0_. Under the association model, labeled *γ* = 1, individuals either have or do not have a pathogenic configuration of alleles and have probabilities of being a case subject *π* and *τ*, respectively. Whether or not an individual has a pathogenic configuration of alleles depends on a function *f* of the genotypes *G*_*i*_ of that individual, a latent binary vector *z* indicating which of the *k* variants are pathogenic, a value *s*_*i*_ equal to the ploidy of the individual at the variant sites, and a variable *m* representing the mode of inheritance governing the disease etiology though the *k* variants:

$$\gamma =0:\mathbb{P}\left(y_{i}=1\right)=\tau_{0},$$

(Equation 1) $$\gamma =1:\mathbb{P}\left(y_{i}=1\right)=\{\begin{array}{cc} \tau & \text{if}\;f\left(G_{i\cdot },z,s_{i},m\right)=0,\\ \pi & \text{if}\;f\left(G_{i\cdot },z,s_{i},m\right)=1. \end{array}$$

The function *f* can represent a dominant inheritance model or a recessive inheritance model that accounts for sex-dependent differences in ploidy on the X chromosome (i.e., X-linked recessive inheritance), depending on variable $m\in \left\{m_{\text{dom}},m_{\text{rec}}\right\}$:

$$f\left(G_{i\cdot },z,s_{i},m_{\text{dom}}\right)=1_{\sum_{j}G_{ij}z_{j}\geq 1},$$

$$f\left(G_{i\cdot },z,s_{i},m_{\text{rec}}\right)=1_{\sum_{j}G_{ij}z_{j}\geq s_{i}}.$$

Thus, the interpretation of *z* depends on the mode of inheritance. In order to have a pathogenic allele configuration, individual *i* requires at least one allele at a variant for which *z*_*j*_ = 1 under a dominant model, but *s*_*i*_ alleles under a recessive model. If genotypes are phased, then a requirement that the *s*_*i*_ pathogenic alleles are on different haplotypes can be imposed. Recent relatedness is a potential confounder because it is correlated with both case/control status and genotype and, therefore, only unrelated individuals should be included in the model.

We place beta priors on all three parameters representing risk of disease:

$$\tau_{0}∼\text{Beta}\left(\alpha_{0},\beta_{0}\right),$$

$$\tau ∼\text{Beta}\left(\alpha_{\tau },\beta_{\tau }\right),$$

$$\pi ∼\text{Beta}\left(\alpha_{\pi },\beta_{\pi }\right).$$

The mean risk of disease for individuals without a pathogenic combination of alleles in the variants under consideration is uncertain under both models, and thus we place uniform priors on *τ*_0_ and *τ* by default. However, as pathogenic combinations of alleles typically confer a high disease risk, we suggest setting the hyperparameters for *π* to $\alpha_{\pi }=6$ and $\beta_{\pi }=1$ (i.e., with a mean of 6/7). However, the prior mean could be adapted, for example, to reflect the consistency with which the disease manifests within families.

We adopt a logistic regression framework for the prior probability that the variants are pathogenic. The logit of the prior probabilities are shrunk toward a common mean, *ω*. If prior information that discriminates between the likely pathogenicity of variants is available, it can be incorporated in the form of a covariate *c* with regression coefficient *ϕ* in the regression equation:

$$z_{j}∼\text{Bernoulli}\left(p_{j}\right),$$

$$\text{logit}\;p_{j}=\omega +\phi c_{j}.$$

One would typically place a Gaussian prior on the intercept *ω* but, for computational purposes, we prefer to use a logit-beta prior with hyperparameters $\alpha_{\omega }$ and $\beta_{\omega }$ (see Appendix A). The prior mean of *ω* should reflect the expected proportion of variants that are pathogenic, conditional on an association, and may depend on the filtering procedures used to select the variants to include in the model. By default, p(ω) reflects a prior expectation that 20% of variants are pathogenic and a prior probability of only 0.01 that the proportion of pathogenic variants exceeds 0.54. This prior is well suited to missense variants but a distribution with a higher mean should be specified if most variants are expected to be pathogenic. This would be the case if the variants under consideration are all protein truncating and thought to be functionally equivalent to each other. To ensure that *ω* can be interpreted as the global mean log odds of pathogenicity, the *c* are required to sum to zero. Thus, any user-supplied weights, ${\tilde{c}}_{j}$, are centered such that $c_{j}={\tilde{c}}_{j}-\left(1/k\right)\sum_{l}{\tilde{c}}_{l}$. We place a log-normal prior on the regression coefficient *ϕ* to force the effects of the *c*_*j*_ to be the same as their signs. The prior mean of *ϕ* is set to 1 so that the *c*_*j*_ are interpretable as prior shifts in the log odds of pathogenicity relative to the mean. A prior variance on *ϕ* of 0.35 ensures that the effect of the co-data can be diminished if the co-data are not informative and increased if they improve the model fit.

Finally, the prior probability on the mode of inheritance parameter *m* and the model indicator parameter *γ* need to be specified. By default, we set the prior probabilities for each mode of inheritance given an association to be the same, i.e., $\mathbb{P}\left(m=m_{\text{dom}}|\gamma =1\right)=0.5$, and we assume that there is only a 1% chance a priori of an association, i.e., ℙ(γ=1)=0.01. However, for a particular set of variants, the choice of values for these parameters could be based on the scientific literature or reference variant databases, for example.

### Inference

The principal quantity of interest is the posterior probability of the model indicator *γ*, which can be derived from a Bayes factor comparing the two models and ℙ(γ). The Bayes factor has two components, the evidence under *γ* = 0 and the evidence under *γ* = 1. A closed-form expression exists for the evidence under either model and it can be computed rapidly under *γ* = 0, irrespective of *y*. However, the expression for the evidence under *γ* = 1 contains a sum over every possible value of *z*, of which there are 2^*k*^, and *k* is usually large enough to render this sum computationally intractable.

To tackle this problem, we reviewed various methods for estimating the evidence of a model^9^ and chose the method of power posteriors,^10^ which enables the evidence to be estimated by Markov chain Monte Carlo (MCMC) sampling. In this method, the MCMC is tempered, which is helpful in a variable selection setting such as ours because it makes exploration of the space of sets of pathogenic variants more efficient. Samples are drawn from a series of related distributions called power posteriors. Each power posterior has a temperature *t* between 0 and 1 and is proportional to the likelihood of the parameters to the power of *t* times the prior. These samples can be combined to obtain an estimate of the integrated likelihood (see Appendix A).

Sampling for our model can be done very efficiently because an MCMC update to *z*_*j*_ entails changes only in $f\left(G_{i\cdot },z,s_{i},m\right)$ for individuals for whom *G*_*ij*_ > 0. For convenience, we estimate the evidence conditional on *m* but we can integrate over it through simple summation. Once the MCMC samples have been collected, the marginal posterior probability of *z* given *γ* and *m* can be obtained directly and used for ranking variants by their likely pathogenicity. The estimated number of pathogenic variants and the expected posterior number of case subjects explained by the pathogenic variants, given *γ* = 1, can also be computed (see Appendix A). The posterior probability of *γ* provides a natural means of ranking sets of variants from different loci across the genome.

The model above assumes that the prior probabilities of variant pathogenicity are conditionally independent. However, particular classes of variants in a locus may confer disease risk, while others may be benign. We can impose a prior correlation structure on the *z* reflecting these competing hypotheses by fitting a different association model for each class of variant. If one of the hypotheses matches the true etiology of disease, then this modeling approach can improve model fit and thus increase power. Let γ∈{1,2,…,g} index the association models and let *I*_*uv*_ indicate whether variant *v* is included in association model *u*. Then, we can compute the probability of association across the competing models as:

$$\mathbb{P}\left(\gamma >0|y,G,c,I\right)=\frac{\sum_{u=1}^{g}\mathbb{P}\left(y|\gamma =u,G^{\left(u\right)},c^{\left(u\right)}\right)\mathbb{P}\left(\gamma =u\right)}{\sum_{u=0}^{g}\mathbb{P}\left(y|\gamma =u,G^{\left(u\right)},c^{\left(u\right)}\right)\mathbb{P}\left(\gamma =u\right)},$$

where $G^{\left(u\right)}=G_{\cdot \left\{v:I_{uv}=1\right\}}$ and $c^{\left(u\right)}=c_{\left\{v:I_{uv}=1\right\}}$. The prior on the model indicator, ℙ(γ), can be informed by external data. For example, if a gene has a high probability of loss-of-function intolerance,^11^ then the prior corresponding to a model of high-impact variants in that gene could be up-weighted relative to competing models. We can also compute the posterior probability of variant pathogenicity averaged over all association models using the following expression:

$$\mathbb{P}\left(z|\gamma >0,y,G,c,I\right)=\frac{\sum_{u=1}^{g}\mathbb{P}\left(z|\gamma =u,y,G^{\left(u\right)},c^{\left(u\right)}\right)\;\mathbb{P}\left(\gamma =u|y,G^{\left(u\right)},c^{\left(u\right)}\right)}{\sum_{u=1}^{g}\mathbb{P}\left(\gamma =u|y,G^{\left(u\right)},c^{\left(u\right)}\right)}.$$

Other quantities of interest, such as the expected posterior number of cases explained by pathogenic variants, can be averaged over models in the same way.

### Simulation Set-Up

We conducted a simulation study under different scenarios and using different methods in order to evaluate power to detect associations, to assess accuracy in variant pathogenicity classification, and to investigate the effect of integration of variant-level co-data on inference. We generated random allele count matrices for 1,000 individuals at *k* rare variant sites with allele frequencies of 0.0017 and 0.03 for the dominant and recessive modes of inheritance, respectively. We used *k* = 25 for the main simulation study. We labeled the first five variants pathogenic and the remaining variants non-pathogenic. The case/control labels were simulated using the expression in Equation 1, assuming *s*_*i*_ = 2 (i.e., diploidy), a particular mode of inheritance (either dominant or recessive), and a particular combination of values for *τ* and π∈{0,(1/10),(2/10),…,1} such that *π* > *τ*. Our selection of *τ* and *π* is comprehensive but for rare diseases we would expect *τ* < 0.5 and *π* ≫ 0.5. For each combination of mode of inheritance, value of *τ*, and value of *π*, 5,000 allele count matrices were generated and 5,000 corresponding case/control vectors were generated. The 5,000 datasets were copied and the case/control labels corresponding to the copied set were permuted to break the association between the genotypes and the phenotype. Thus, under each scenario, we had a pool of 10,000 datasets, of which half were generated under a model of association and half were generated under a model of no association.

In order to assess the performance of different methods in a realistic setting, we evaluated their ability to rank non-permuted datasets among a large set of permuted datasets. Under each simulation scenario, we generated mixtures of 10 non-permuted and 990 permuted datasets selected at random from the corresponding pool. We then applied each method and computed the mean positive predictive value (PPV), over 10,000 repetitions, at 80% power. The PPV, which is equal to one minus the false discovery rate (FDR), is inversely related to power. Thus, a higher PPV for a given power implies greater power for a given FDR. We preferred to evaluate PPV for a given power rather than power for a given FDR because empirical power changes monotonically as the rank threshold for declaring a positive result is lowered, while the empirical FDR does not.

We selected the methods ADA, CAST, and SKAT for comparison as they represent diverse approaches: ADA enables individual variant-level inference, CAST is based on the popular Burden test but can account for either dominant or recessive inheritance modes, and SKAT is a popular and flexible method designed for rare variants affecting complex traits. The default linear kernel function for SKAT is used here. The other methods mentioned above were either inapplicable (e.g., vbdm requires a continuous response), unavailable (BRVD), or shown to be inferior to ADA in a previous publication.^12^ Note that ADA p values were computed using 10,000 permutations instead of the default 1,000 in order to reduce the number of ties (parts of the ADA code were re-implemented in C++ in order to complete our simulation study in a reasonable amount of time; modified code available on request).

The results were ranked based on the posterior probability that *γ* = 1 for BeviMed and the negative log p value of association for the other methods. Variants were ranked according to BeviMed’s marginal posterior probability for the components of *z* and according to inclusion in ADA’s variant selection. The other methods do not provide variant-level inference. Although the backward elimination procedure is implemented for SKAT, it is so slow as to make its use impractical in even a moderately sized study such as this.

To demonstrate the effect of including prior information regarding variant pathogenicity on BeviMed’s inference, we conducted a further study whereby we simulated datasets with *m* = *m*_dom_, *τ* = 0.2, and *π* = 0.85 and modified the values of the variant-specific co-data ${\tilde{c}}_{j}$ as follows. The values of ${\tilde{c}}_{j}$ for all variants was set to either 1 or 0. The number of truly pathogenic variants that were assigned the value 1 was set to 0, 1, 2, 3, 4, or 5, and the number of truly non-pathogenic variants that were assigned the value 1 was set to 0, 4, 8, 12, 16, or 20. Thus, the proportions of correctly and incorrectly up-weighted variants were varied between 0 and 1 in increments of 0.2. In the extreme, the co-data could support the true classification exactly or support the inverted classification exactly. As SKAT can incorporate variant-specific relative weights, we applied it to the same simulated data, setting SKAT’s weights for up-weighted variants to twice that of the others. This choice of up-weighting factor was as low as possible while ensuring that, when the weights were perfectly concordant with the true pathogenicity of the variants, the PPV was approximately the same for SKAT as for BeviMed. Expected PPV at 80% power was estimated as described above, based on 5,000 truly associated and 5,000 permuted datasets, for BeviMed and SKAT under each combination of proportions of correctly up-weighted and incorrectly up-weighted variants.

### Application to Real Data

The NIHR BioResource–Rare Diseases has generated whole-genome sequencing data for 6,586 unrelated individuals with diverse rare diseases in an effort to identify novel genetic etiologies. We applied BeviMed to the data, setting the case/control status based on two dichotomous phenotypes represented in the study: pathologically low numbers of platelets in the blood stream (thrombocytopenia) with absence of syndromic features $\left(\sum_{i}y_{i}=184\right)$ and Roifman syndrome $\left(\sum_{i}y_{i}=3\right)$.

Hereditary thrombocytopenia can be caused by variants in a large number of genes with diverse functions, including genes encoding transcription factors, cytoskeletal proteins, and membrane proteins.^13^ Severe thrombocytopenia is typically monogenic and non-syndromic forms are usually dominant. Roifman syndrome (MIM: 616651) is a rare autosomal-recessive disease with symptoms including growth retardation, spondyloepiphyseal dysplasia, and cognitive delay, initially described by Roifman et al.^14^ Last year, variants in the non-coding gene *RNU4ATAC* (MIM: 601428) were identified as the cause of this disease on the basis of pedigree studies involving six case subjects.^15^ Within the bleeding and platelet disorders branch of the NIHR BioResource dataset, three unrelated case subjects with Roifman were enrolled because they presented with immune thrombocytopenia.

For each gene, we considered single-nucleotide variants (SNVs) and short insertions/deletions (indels) with an allele frequency in ExAC^11^ and the whole-genome sequencing component of UK10K^16^ less than 1/1,000 and large deletions overlapping exons with an internal frequency less than 1/1,000. SNVs and indels had to have a HIGH or MODERATE Variant Effect Predictor (VEP)^17^ impact or they had to have a VEP Sequence Ontology-coded consequence that included non_coding_transcript_exon_variant, 5_prime_UTR_variant, or 3_prime_UTR_variant. If a variant had consequences in relation to multiple transcripts of the same gene, only the highest-impact consequence was retained. In total, we considered 1,338,830 variants in 35,205 gene loci, each containing between 1 and 2,615 variants.

We set $\mathbb{P}\left(m=m_{\text{dom}}|\gamma =1\right)$ to 0.8 for thrombocytopenia and 0.1 for Roifman syndrome, to reflect the belief that these diseases tend to be dominantly and recessively inherited, respectively. For each locus, we considered four association models corresponding to four classes of variants:

• High: large deletions and variants with a HIGH impact annotation
• Moderate: variants with a MODERATE or HIGH impact annotation or a consequence including non_coding_transcript_exon_variant but none of the UTR-related consequences
• 5′ UTR: variants without a MODERATE or HIGH impact annotation and a consequence including 5_prime_UTR_variant
• 3′ UTR: variants without a MODERATE or HIGH impact annotation and a consequence including 3_prime_UTR_variant

The hyperparameters were assigned default values except for $\alpha_{\omega }$ and $\beta_{\omega }$, which we set to (2,1) instead of (2,8) under the “high” model. This reflects a belief that a greater proportion of variants are likely to be pathogenic under the high model than under the other three models. When we fitted the “moderate” model, we up-weighted the variants that were also included in the high class relative to the others by setting their uncentered weights ${\tilde{c}}_{j}$ to 1 rather than 0. For coding loci, we assigned prior probabilities of 0.004, 0.003, 0.002, and 0.001 to the four models above, respectively, in order to reflect the relative biological plausibility of the different classes of variants being involved in disease. For non-coding genes, we assigned a prior probability of the moderate model equal to 0.01. Thus, ℙ(γ>0)=0.01 for all genes. For completeness, we also applied SKAT to the four classes of variants described above separately, using default settings, and retained the result with the lowest p value for each locus.

## Results

### Simulation Study

**Figure 1:**
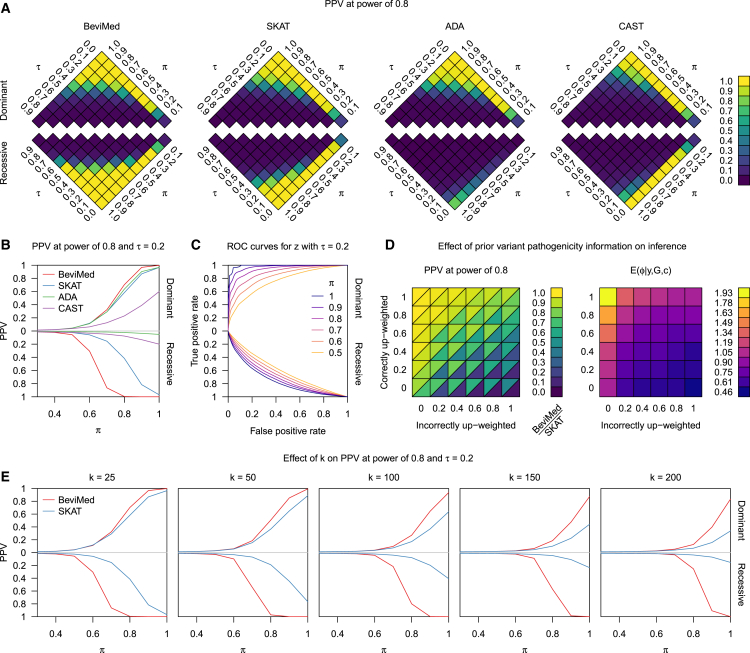
Simulation Study (A and B) Results of the simulation study. Mean PPV at power of 80% over repeat simulation of the BeviMed, SKAT, ADA, and CAST rare variant association tests for data simulated using the expression in Equation 1 for various combinations of values of *τ* and *π*. (C) Receiver operating characteristic (ROC) curves for the classification of variants as pathogenic by BeviMed for different values of *π*. (D) Left: mean PPV at power of 80% for BeviMed and SKAT at *τ* = 0.2 and *π* = 0.85, for varying proportions of pathogenic and non-pathogenic variants being up-weighted in the co-data variables. Right: posterior mean of *ϕ* corresponding to the applications of BeviMed on the left-hand grid. (E) Mean PPV at power of 80% over repeat simulation of the BeviMed and SKAT association tests for different values of *k*.

Under a dominant model, BeviMed had a slightly higher PPV than the other methods while, under a recessive model, it greatly outperformed competing methods: when *π* = 0.8 and *τ* = 0.2, BeviMed had a PPV of 100%, while SKAT, CAST, and ADA had a PPV of 42%, 8%, and 2%, respectively (Figures 1A and 1B). This favorable performance was achieved despite using the same priors for model parameters *τ* and *π*, irrespective of the values of *τ* and *π* used to simulate the data. We note that BeviMed’s performance for *τ* = 0.2 was approximately the same for the following three pairs of values for the hyperparameters $\alpha_{\omega }$ and $\beta_{\omega }$: (2,8), which is the default, (1,1), which places a uniform prior on expit(ω), and (2,1), which places higher prior weight on values of expit(ω) near 1.

For *τ* = 0.2 and high *π*, BeviMed was able to provide accurate rankings of variants by estimated pathogenicity, particularly under a dominant mode of inheritance (area under the curve = 0.97 at *π* = 0.9, Figure 1C). ADA’s average classification of variant pathogenicity at *π* = 0.9 gave a true positive rate of 0.78 and a false positive rate of 0.063, while BeviMed’s true positive rate at that same false positive rate was 0.88.

The results of the simulation study assessing the effect of incorporating variant weights show that BeviMed is substantially more robust to deleterious weightings (Figure 1D, left). When the co-data matched the truth perfectly, the power for BeviMed and SKAT was approximately the same (by design), but when the co-data was entirely counter-productive, BeviMed’s PPV was 0.46 and SKAT’s PPV was 0.06. BeviMed’s advantage was achieved naturally in our Bayesian setting through modulation of *ϕ*, which had a posterior expectation of 1.93 when the co-data was most useful but only 0.46 when it was least useful (Figure 1D, right).

We evaluated the performance of BeviMed in relation to the most competitive alternative method, SKAT, using the same parameters described above, but increasing the total number variants *k* to 50, 100, 150, and 200. Power decreased for both methods as the total number of variants increased, but the discrepancy in power between BeviMed and SKAT increased (Figure 1E). For example, under the dominant model, BeviMed’s PPV at *k* = 200 and *π* = 1.0 was 83% while SKAT’s PPV was only 34%.

### Computational Performance

We compared the execution times of the different association tests, including SKAT with backward elimination, on simulated datasets generated as described above using N∈{1000,5000,100000}, k∈{25,100,1000}, and allele frequency of 1/1,000. The results, displayed in Table 1, show that CAST is the fastest method, as it uses a straightforward Fisher’s exact test. However, CAST is substantially less powerful than BeviMed under both dominant and recessive models (Figure 1). BeviMed has comparable execution time to SKAT for small datasets and surpasses it for large datasets, as BeviMed’s complexity scales with $\sum_{i,j}G_{ij}>0$, which typically increases only linearly with *N*. SKAT was also run using the other kernels available in the R package: identity by state, quadratic, and two-way interactions. All three modes were substantially slower than BeviMed and linear SKAT (Table 1), less powerful than BeviMed in the simulation study, and less powerful than linear SKAT under at least one mode of inheritance. BeviMed has vastly superior performance to the other methods which can infer the pathogenicity of variants, while also reporting posterior uncertainty in pathogenicity status. The complete set of applications to the data from the NIHR BioResource, which comprises 2 phenotypes, 35,205 loci, 2 modes of inheritance, and 4 variant classes, took 7 hr to complete using 16 CPU cores.

**Table 1:** Performance Comparison

**Method**	**N = 1,000, k = 25**	**N = 1,000, k = 100**	**N = 5,000, k = 25**	**N = 5,000, k = 100**	**N = 100,000, k = 1,000**
**Association Tests with Variant Identification**					

BeviMed	0.03	0.09	0.07	0.23	38.90
ADA	3.69	11.30	18.76	59.23	–
BE-SKAT	53.46	175.18	137.39	799.80	–

**Association Tests without Variant Identification**					

CAST	0.01	0.01	0.02	0.05	1.77
SKAT	0.02	0.05	0.09	0.30	140.82
SKAT (IBS)	3.73	4.38	548.47	675.62	–
SKAT (quadratic)	4.08	4.12	571.69	598.96	–
SKAT (2wayIX)	4.09	4.37	580.90	575.67	–

Execution times in seconds of different association tests for datasets with different *N* and *k*. BE-SKAT refers to SKAT with backward elimination of variants. SKAT (IBS), SKAT (quadratic), and SKAT (2wayIX) refer to application of SKAT using the weighted identity by state, quadratic, and two-way interactions kernel functions, respectively. The p values for ADA and BE-SKAT were computed using their default number of permutations, respectively 1,000 and 300. Dashes indicate that the method took longer than 1 hr to run.

### Identifying Associations with Thrombocytopenia

**Figure 2:**
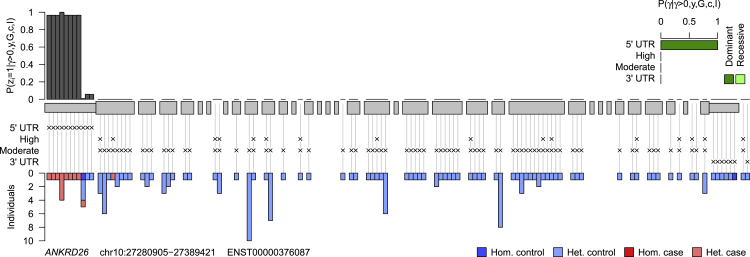
Posterior Probability of Pathogenicity for Rare Variants in *ANKRD26* Results obtained by applying our inference procedure to rare allele counts in *ANKRD26* against the thrombocytopenia case/control label. Exons are represented by gray blocks starting from the 5′ UTR on the left and ending with the 3′ UTR on the right. The classes that each variant belongs to are indicated by crosses. The bar chart in the top right shows the posterior probability of each association model under each mode of inheritance conditional on an association being present at the locus. The gray bars above show the marginal posterior probabilities of pathogenicity for individual rare variants conditional on an association being present at the locus. The inference algorithm was run with 100,000 iterations instead of the usual 1,000 in order to reduce jitter due to Monte Carlo sampling error. The bar chart beneath shows the breakdown of heterozygous and homozygous carriers of the variants between case and control subjects.

The median value of the posterior probability of association with thrombocytopenia across all gene loci was 0.0064. The independent gene loci for which the posterior probability of association exceeded 0.9 are shown in Table 2. We show the posterior probability of association, the posterior probability of the mode of inheritance parameter, the estimated number of case subjects explained by the pathogenic variants, the estimated number of pathogenic variants that are present in the case subjects, and the total number of variants considered. These results corroborate established knowledge of platelet disorders. *ACTN1* (MIM: 102575)-related macrothrombocytopenia is a dominant bleeding and platelet disorder recognized since 2013.^18^ *GP1BB* (MIM: 138720) has traditionally been linked to a recessive bleeding and platelet disorder called Bernard-Soulier syndrome,^19^ but earlier this year we reported a dominant mode of inheritance resulting in a milder platelet phenotype.^20^ The posterior on the mode of inheritance parameter strongly favored dominance in this case, which is consistent with an absence of Bernard-Soulier-affected case subjects in our dataset. *RUNX1* (MIM: 151385) encodes a transcription factor linked with a dominant platelet disorder with associated myeloid malignancy. *MYH9* (MIM: 160775) harbors variants responsible for *MYH9*-related disorder, which is characterized by macrothrombocytopenia and occasional Döhle-like inclusion bodies in neutrophils and pathologies of the ear, eye, kidney, or liver. Finally, variants in the 5′ UTR of *ANKRD26* (MIM: 610855) were reported to result in non-syndromic macrothrombocytopenia in 2011^21^ after an initial erroneous report that variants in the neighboring gene *ACBD5* (MIM: 616618) were responsible.^22^ The association we have identified is driven by variants in the 5′ UTR, despite this class of variants being down-weighted relative to the classes comprising variants with missense or high-impact predicted consequences on the translated gene product. The variant level results of the inference shown in Figure 2 indicate the high posterior probability of association for the first eight variants in the 5′ UTR. It is noteworthy that one of the variants, which encodes c.−113A>C and was reported in a follow-up^23^ to the original 2011 paper, does not appear to be pathogenic, as five out of the six individuals with the variant, including one homozygous for the alternate allele, do not have a bleeding or platelet disorder.

**Table 2:** Independent Loci Having ℙ(γ=1|y)>0.9

**Locus**	**Posterior Probability of Association**	**Posterior Probability of Dominance**	**Modal Model**	**Estimated Number of Explained Case Subjects**	**Estimated Number of Explaining Variants**	**Number of Variants**
*ANKRD26*	1.000	1.000	5′ UTR	10.792	7.792	87
*RUNX1*	1.000	1.000	moderate	8.153	8.191	214
*MYH9*	1.000	1.000	moderate	10.964	9.116	141
*GP1BB*	0.999	1.000	moderate	8.223	7.228	69
*ACTN1*	0.999	1.000	moderate	9.867	7.867	121

Independent loci with a posterior probability of association with thrombocytopenia greater than 0.9.

There were four additional loci having ℙ(γ=1|y)>0.9. They all tagged a true association listed in Table 2 but were labeled with the names of neighboring genes and had lower posterior probabilities of association. A missense variant in *WAC* (MIM: 615049) was in linkage disequilibrium with one of the 5′ UTR variants in *ANKRD26*, inducing ℙ(γ=1|y)=0.993 for the *WAC* locus. The other three results were induced by the presence of deletions in *RUNX1* spanning three neighboring RNA genes or pseudogenes: *AF015262.2* (ℙ(γ=1|y)=0.978), *RPL34P3* (ℙ(γ=1|y)=0.976), and *EZH2P1* (ℙ(γ=1|y)=0.974).

The alternate method that was most powerful based on the results of the simulation, SKAT, did not rank the loci listed above as highly, even when only the variant class with the lowest p value was retained for each gene. *RUNX1*, *ANKRD26*, *MYH9*, *ACTN1*, and *GP1BB* had ranks of 1, 3, 8, 16, and 74, respectively, with none of the other loci in the top 20 ranks being known to be implicated in thrombocytopenia.

### Identifying Variants Responsible for Roifman Syndrome

**Figure 3:**
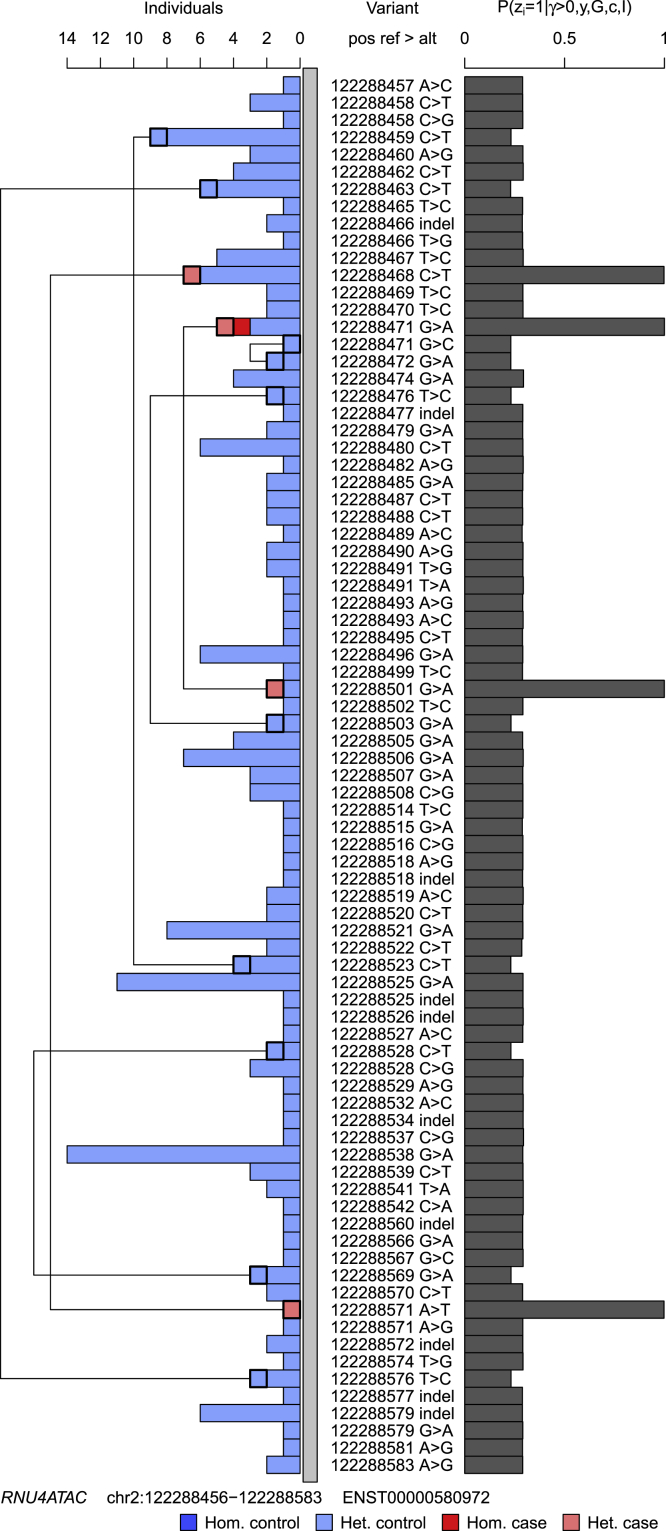
Posterior Probability of Pathogenicity for Rare Variants in *RNU4ATAC* Results of applying the inference procedure to rare allele counts in *RNU4ATAC* against the Roifman syndrome case label. The bar chart on the right shows the marginal posterior probabilities of pathogenicity for each rare variant conditional on an association being present at the locus. The inference algorithm was run with 100,000 iterations instead of the usual 1,000 in order to reduce jitter due to Monte Carlo sampling error. The bar chart on the left shows the breakdown of heterozygous and homozygous carriers of the variants in case and control subjects. Compound heterozygous individuals with two rare alleles in *RNU4ATAC* were observed, and for each such individual a line is drawn linking the two variants.

The locus with the highest posterior probability of association with the Roifman syndrome case label was *RNU4ATAC* (ℙ(γ=1)=1.000), driven by four different single-nucleotide variants in this non-coding gene. Two of the case subjects were compound heterozygous, including for a variant observed in six control subjects, and one was homozygous. As all but one of the variants were seen only in heterozygosity, the posterior probability of variant pathogenicity conditional on recessive inheritance was relatively high across the gene but markedly lower than the causal variants observed in the case subjects, which had a posterior probability of pathogenicity very close to 1 (Figure 3). All other genes had a posterior probability of association less than 0.9 and the expected number of case subjects explained by the variants in other loci was less than 2. SKAT assigned *RNU4ATAC* a p value of 0, but this was also the case for 34 other genes, which were tied in the top rank.

## Discussion

We have presented a Bayesian genetic association method for rare diseases that is more powerful than existing methods, particularly for the recessive mode of inheritance, and provides summary statistics on variant-level pathogenicity and mode of inheritance very efficiently. It enables mode of inheritance to be integrated out or inferred from the data. Indeed, we were able to determine a dominant mode of inheritance for variants in a gene, *GP1BB*, that has been associated only with a recessive disorder for more than 30 years. Given an association under a particular mode of inheritance, our method also estimates the number of case subjects explained by pathogenic variants and the number of variants that are pathogenic.

Prior information specific to a particular set of variants under consideration can modulate the evidence of association, which can be critical when the number of case subjects with a shared genetic etiology is small. For example, the prior on the model indicator *γ* can be adjusted to reflect locus-specific genomic and epigenomic knowledge in order to encourage regions with higher prior plausibility of involvement in the disease phenotype to rank more highly than if the same prior had been used across all regions. The prior probability of pathogenicity for a particular variant, given the association model, can be modulated by knowledge about the variant, such as predicted consequence, allele frequency, or conservation. These variant weights are interpretable as prior shifts in the log odds of pathogenicity, which provides an intuitive basis for assigning particular values to them. In sharp contrast to frequentist approaches, we use a flexible prior on the effect sizes of the weightings that reflects the uncertainty in their utility.

The results of inference on different subsets of rare variants in a locus (selected, for example, on the basis of their predicted consequences) can be interpreted and combined easily in a Bayesian framework using a model selection procedure. The posterior probability of variant pathogenicity and other quantities of interest can be averaged over models. In addition to increasing statistical power if particular classes of variants in a locus are the only ones that confer disease risk, this feature also allows inference of the kind of variants responsible for disease, which may suggest particular genetic etiologies. In our applications, we were able to identify a set of variants in the 5′ UTR of a gene that causes a platelet disorder. The high posterior probability of pathogenicity of variants in the 5′ UTR to the exclusion of coding variants, even those observed only in case subjects, was made possible by our model selection procedure.

Variants highlighted by a method such as ours would usually undergo assessment by a multidisciplinary diagnostic team and it would resolve increasing numbers of case subjects over time. In our application to real data, we have kept the case/control labels the same for each application. However, in the context of genetically heterogeneous diseases, we would recommend relabelling any case subject whose phenotype has been fully accounted for by pathogenic or likely pathogenic variants in a different locus as a control. This boosts specificity as it makes it less likely for a non-pathogenic rare variant carried by a case to induce a high probability of association.

The model assumes that relatedness between individuals is sufficiently low as not to be associated with either case/control status or the genotypes. In practice, we recommend removal of any first, second, or third degree relatives. Our method is designed to be applied to up to thousands of rare variants at a time and efforts should be made to ensure all potentially implicated variants in a locus are included in the model, or the set of models, under comparison. Rare variants would typically be unlinked within a locus but may occasionally be linked across loci. For example, large deletions may span multiple genes and certain pairs of rare variants could be in linkage disequilibrium. In these situations, a non-pathogenic rare variant in one locus linked to a pathogenic variant in another locus could induce a non-causal association. Such associations can either be filtered post hoc through comparison of inference results in nearby loci or avoided altogether by joint modeling of variants across multiple nearby loci.

Although Bayesian inference is typically thought of as slow, our implementation can handle data from more than a million variants spread across tens of thousands of regions called in thousands of samples in a few hours. BeviMed is thus capable of handling with ease the demands of modern genomic datasets in the coding and the regulatory regions of the genome.

## Appendix A

Inference on presence of an association is based on the posterior probability of the model indicator *γ*, which can be derived from the evidence under each model and the prior on *γ*:

$$\mathbb{P}\left(\gamma =1|y\right)=\frac{\mathbb{P}\left(y|\gamma =1\right)\mathbb{P}\left(\gamma =1\right)}{\sum_{u\in \left\{0,1\right\}}\mathbb{P}\left(y|\gamma =u\right)\mathbb{P}\left(\gamma =u\right)}.$$

The evidence for the baseline model, ℙ(y|γ=0), can be computed efficiently using the beta function:

$$\frac{B\left(\alpha_{0}+\sum_{i}y_{i},\beta_{0}+N-\sum_{i}y_{i}\right)}{B\left(\alpha_{0},\beta_{0}\right)}.$$

The evidence for the association model, ℙ(y|γ=1), can be expressed by conditioning on the different modes of inheritance and summing:

$$\mathbb{P}\left(y|\gamma =1\right)=\mathbb{P}\left(y|\gamma =1,m=m_{\text{dom}}\right)\mathbb{P}\left(m=m_{\text{dom}}|\gamma =1\right)+\mathbb{P}\left(y|\gamma =1,m=m_{\text{rec}}\right)\mathbb{P}\left(m=m_{\text{rec}}|\gamma =1\right),$$

where ℙ(y|γ=1,m) is given by:

$$\sum_{z\in {\left\{0,1\right\}}^{k}}\int_{-\infty }^{\infty }\int_{-\infty }^{\infty }p\left(y|z,m\right)p\left(z|\omega ,\phi \right)p\left(\omega \right)p\left(\phi \right)\;\text{d}\omega \;\text{d}\phi .$$

For brevity, we have omitted the hyperparameters $\alpha_{\tau }$, $\beta_{\tau }$, $\alpha_{\pi }$, and $\beta_{\pi }$ from the conditioning in p(y|z,m) above.

The likelihood p(y|z,m) factorizes into two components corresponding to individuals with and without a pathogenic combination of alleles, where the rate parameters *τ* and *π* can be integrated out analytically. Thus the likelihood can be expressed in closed form:

(Equation A1) $$\frac{B\left(\alpha_{\tau }+\sum_{i}y_{i}\left(1-x_{i}^{\left(z\right)}\right),\beta_{\tau }+\sum_{i}\left(1-y_{i}\right)\left(1-x_{i}^{\left(z\right)}\right)\right)}{B\left(\alpha_{\tau },\beta_{\tau }\right)}\times \frac{B\left(\alpha_{\pi }+\sum_{i}y_{i}x_{i}^{\left(z\right)},\beta_{\pi }+\sum_{i}\left(1-y_{i}\right)x_{i}^{\left(z\right)}\right)}{B\left(\alpha_{\pi },\beta_{\pi }\right)},$$

where $x_{i}^{\left(z\right)}=f\left(G_{i\cdot },z,s_{i},m\right)$.

As noted in the main text, we use a logit-beta prior on *ω*, that is:

$$\text{expit}\left(\omega \right)∼\text{Beta}\left(\alpha_{\omega },\beta_{\omega }\right).$$

Thus, when $c_{j}=0\;\forall j\in \left\{1,\ldots ,k\right\}$, *z* is independent of *ϕ*, and both *ω* and *ϕ* can be integrated out:

$$p\left(z\right)=\frac{B\left(\alpha_{\omega }+\sum_{j}z_{j},\beta_{\omega }+k-\sum_{j}z_{j}\right)}{B\left(\alpha_{\omega },\beta_{\omega }\right)}.$$

By default, $\alpha_{\omega }=2$ and $\beta_{\omega }=8$. The space of *z* grows exponentially with the number of variants *k*, which can run into the dozens or hundreds. Therefore, despite the formulation of the model enabling many of the parameters to be integrated out analytically, the expression for the integrated likelihood cannot be evaluated in practice. Below we describe an alternative method to estimate the integrated likelihood which is computationally tractable.

The method of power posteriors^10^ allows us to estimate ℙ(y|γ=1,m) by sampling from $\mathbb{P}{\left(z|y,m\right)}^{t}\mathbb{P}\left(z\right)$ at temperatures $t\in \left\{t_{0}=0,\ldots ,t_{L}=1\right\}$ using MCMC. Let ${\tilde{z}}_{l}^{\left(b\right)}$ be the *b*th sample drawn at temperature $t_{l}$. The log integrated likelihood logℙ(y|γ=1,m) can then be estimated by:

(Equation A2) $$\sum_{l=0}^{L-1}\text{log}\left(\frac{1}{\left|{\tilde{z}}_{l}\right|}\sum_{b=1}^{\left|{\tilde{z}}_{l}\right|}e^{\left(t_{l+1}-t_{l}\right)\;\text{log}\;\mathbb{P}\left(y|{\tilde{z}}_{l}^{\left(b\right)},\;m\right)}\right).$$

Running Markov chains at different temperatures concurrently allows exchanges of state between chains at adjacent temperatures, which encourages good mixing. If the Kullback-Leibler (KL) divergence between adjacent power posterior distributions is large, the resulting estimates of ℙ(y|γ=1,m) may be susceptible to substantial numerical error. The method that minimizes this error involves tuning the temperatures using a procedure such as interval bisection^24^ and subsequently re-generating the chains to allow mixing between them. By default we use a pre-selected set of temperatures $t={\left(l/6\right)}^{2}$ for l∈{0,1,…,6}, and draw 1,000 samples from each chain. This works well in practice and avoids the need to discard an initial set of MCMC samples for tuning the temperatures.

The use of MCMC to tackle this overall inference problem is in contrast to other methods designed for similar purposes,^12^, ^25^ probably because of the stringent requirements for computational speed. However, our algorithm contains features that makes MCMC sampling efficient.

In each chain, Gibbs sampling is used to update each individual component of *z* in turn. An update to *z*_*j*_ consists of sampling from its full conditional distribution:

$$\text{Bernoulli}({\left(1+\frac{p\left(y|z^{\left(0\right)},m\right)p\left(z^{\left(0\right)}|\omega ,\phi \right)}{p\left(y|z^{\left(1\right)},m\right)p\left(z^{\left(1\right)}|\omega ,\phi \right)}\right)}^{-1}),$$

where $z_{j^{\prime }}^{\left(0\right)}=z_{j^{\prime }}^{\left(1\right)}=z_{j^{\prime }}$ for $j^{\prime }\neq j$ and $z_{j}^{\left(0\right)}=0,z_{j}^{\left(1\right)}=1$. During the course of the algorithm, we keep track of $x_{i}=f\left(G_{i\cdot },z,s_{i},m\right)$ for each individual. Given an update of a single component of *z*, only individuals for whom *G*_*ij*_ > 0 need to have their corresponding value of *x*_*i*_ updated. *G* is often sparse as it typically represents rare allele counts, allowing this operation to be performed quickly. If values for *c* are specified, then *ω* and *ϕ* are updated using a Metropolis Hastings within Gibbs.

Averaging over the space of all variant/variable sets using MCMC is a daunting challenge, in particular in circumstances such as these where non-additive models for the interaction effects of the variables are used. However, in practice, there is little collinearity between rare variant allele counts in unrelated individuals, rare allele count matrices are sparse, and the interaction effects in dominant and recessive inheritance are quite simple, leading to low correlation between the elements of *z*. This means that the sampling procedure can explore the space of *z* efficiently.

However, when *k* is large and the mode of inheritance is recessive, with some case subjects being compound heterozygous, mixing of the MCMC sampler could potentially be poor if only one element of *z* is updated at a time. In particular, it could be very rare for the Markov chain to transition from a state satisfying $z_{j_{1}}=z_{j_{2}}=0$ for some truly pathogenic variants *j*_1_, *j*_2_, to a state where $z_{j_{1}}=z_{j_{2}}=1$, as there may be no intermediate state that would lead to an increase in likelihood. This is particularly problematic if the prior on *ω* is concentrated near 0. Thus, under *m* = *m*_rec_, we propose updates to elements of *z* corresponding to variants occurring in the same individuals in tandem, which overcomes the potential rarity of sampling a state with a high likelihood.

The likelihood shown in Equation A1 can be expressed in terms of ratios of gamma functions with arguments that differ by integer amounts less than or equal to the number of individuals. Hence, the differences between all possible return values of log Γ that are required by the procedure can be computed before commencing the sampling and stored to avoid evaluating the log Γ function repeatedly. Evaluating log Γ is the computational bottleneck in the Markov chain updates, and replacing it with look-ups in the pre-computed values tables results in significant speed-ups.

To further improve computational efficiency while maintaining adequate precision, the implementation provides an option to stop sampling once the estimated evidence lies within a given confidence interval, or once there is sufficient confidence that the log evidence is below a given threshold. This behavior is implemented based on the method of consistent batch means.^26^ The log evidence is the sum of the logarithms of expectations taken with respect to the power posteriors (Equation A2), so the central limit theorem does not apply and we estimate the confidence interval by simulation.

The samples drawn from the MCMC routine at the temperature *t* = 1 can be used to compute the expected number of case subjects whose risk was due to their pathogenic configuration of alleles, $E_{z|y}\sum_{i}y_{i}x_{i}^{\left(z\right)}$, and the expected number of variants involved in the explanation, $E_{z|y}\sum_{j\in \left\{j^{\prime }:\sum_{i}G_{ij^{\prime }}y_{i}x_{i}^{\left(z\right)}>0\right\}}z_{j}$.

## Web Resources

ADA, http://homepage.ntu.edu.tw/∼linwy/ADAprioritized.html
BE-SKAT, http://www.columbia.edu/∼ii2135/Software_BE_HM.zip
BeviMed, https://cran.r-project.org/web/packages/BeviMed/
OMIM, http://www.omim.org/
SKAT, https://cran.r-project.org/web/packages/SKAT/

## Supplemental Data

Table S1. Additional Members and Collaborators of the NIHR BioResource
